# The personal and clinical impact of screen-detected maternal rheumatic heart disease in Uganda: a prospective follow up study

**DOI:** 10.1186/s12884-020-03189-z

**Published:** 2020-10-09

**Authors:** Sonia Voleti, Emmy Okello, Meghna Murali, Rachel Sarnacki, Albert Majwala, Renny Ssembatya, Olivia Bakka, Henriator Namisanvu, Angela Njeri, Alphonsus Matovu, Kristen DeStigter, Craig Sable, Andrea Beaton

**Affiliations:** 1 Department of Cardiology, Children’s National Medical Center, 111 Michigan Avenue NW, Washington, DC 20010 USA; 2 The Uganda Heart Institute, Ward 1 C, Mulago Hospital Complex, Kampala, Uganda; 3 Imaging the World Africa, Naayla-Namugongo Rd, Naayla, Uganda; 4 Mubende Regional Referral Hospital, Mubende, Uganda; 5 University of Vermont Medical Center, 111 Colchester Avenue, Burlington, VT USA; 6 Cincinnati Children’s Hospital Medical Center, 3333 Burnet Avenue, Cincinnati, OH USA; 7 The University of Cincinnati School of Medicine, Cincinnati, OH USA

**Keywords:** Rheumatic heart disease, Maternal health, Uganda, Health disparities, Echocardiography, Screening

## Abstract

**Background:**

Pre-existing maternal cardiac disease is a significant contributor to adverse maternal, fetal, and neonatal outcomes. In 2015–2017, our team conducted the first community-based study of maternal rheumatic heart disease (RHD) in sub-Saharan Africa and identified RHD in 88% of those with pre-existing heart disease.

Here we conducted a follow up investigation of women previously identified with RHD, describing clinical and echocardiographic outcomes, identifying barriers to medical adherence and evaluating the personal impact of RHD.

**Methods:**

A 2 week prospective follow up was completed at sites in Central and Eastern Uganda. Participants underwent a three-step mixed methods study comprising of 1) direct structured interview targeting clinical history and medication adherence, 2) echocardiogram to evaluate left-sided heart valves, and 3) semi-structured guideline interview to elicit personal impacts of RHD.

**Results:**

The team evaluated 40 (80%) of the original 51 mothers with RHD at a median post-partum time of 2.5 years after delivery (IQR 0.5). Echocardiographic data showed improvement in nine women with the remaining 31 women showing stable echocardiographic findings. Adherence to Benzathine penicillin G (BPG) prophylaxis was poor, with 70% of patients either poorly adherent or non-adherent.

Three major themes emerged from interviews: 1) social determinants of health (World Health Organization, Social determinants of health, 2019) negatively affecting healthcare, 2) RHD diagnosis negatively affecting female societal wellbeing, 3) central role of spouse in medical decision making.

**Conclusions:**

Screening echocardiography can identify women with pre-existing rheumatic heart disease during pregnancy, but long-term follow-up in Uganda reveals adherence to medical care following diagnosis, including BPG, is poor. Additionally, mothers diagnosed with RHD may experience unintended consequences such as social stigmatization. As identification of occult RHD is critical to prevent adverse pregnancy outcomes, further research is needed to determine how to best support women who face a new diagnosis of RHD, and to determine the role of screening echocardiography in high-risk settings.

## Background

Pre-existing maternal cardiac disease is a major contributor to adverse maternal, fetal, and neonatal outcomes. While the reach is global, the prevalence and impact of disease is highest in low- and middle-income countries (LMICs), where 88–90% of maternal heart disease is rheumatic heart disease (RHD) [1]. While outcomes for women living with RHD in well-resourced settings have been described [2, 3] data are sparser for women living in low-resource settings.

In 2015–2017, our team conducted the first community-based study of maternal RHD in sub-Saharan Africa. Echocardiographic screening (2012 World Heart Federation criteria [4]) of over 3500 women in 2 rural districts revealed 1.7% of women had preexisting heart disease, 88% of which was definite RHD [4]. Only 3% of women with heart disease were aware of diagnosis prior to screening, yet slightly over half were found to be symptomatic during pregnancy necessitating medical management, referral to higher-level facilities, or changes in delivery planning. Even after diagnosis, the percent attributable risk of heart disease to the maternal, fetal and neonatal mortality was high: 11, 1.1, and 6.0% respectively [5].

While the immediate consequences of maternal rheumatic heart disease are of critical importance, they do not fully describe how RHD affects women living in low-resource settings. Survivors may face stigmatization in their families and communities, experience difficulties accessing ongoing care, and face difficult future reproductive decisions [6]. Furthermore, RHD can worsen with pregnancy, and more data are needed about longitudinal clinical outcomes to inform medical management and patient counseling.

Here, we report long term follow-up study of women diagnosed with RHD in pregnancy. Our aims were to 1) describe long term clinical and echocardiographic outcomes, 2) identify barriers to medical access and medication adherence, and 3) evaluate the personal impact of disease. These data provide a more holistic view of the challenges faced by mothers living with RHD.

## Methods

### General methods

**Fig. 1:**
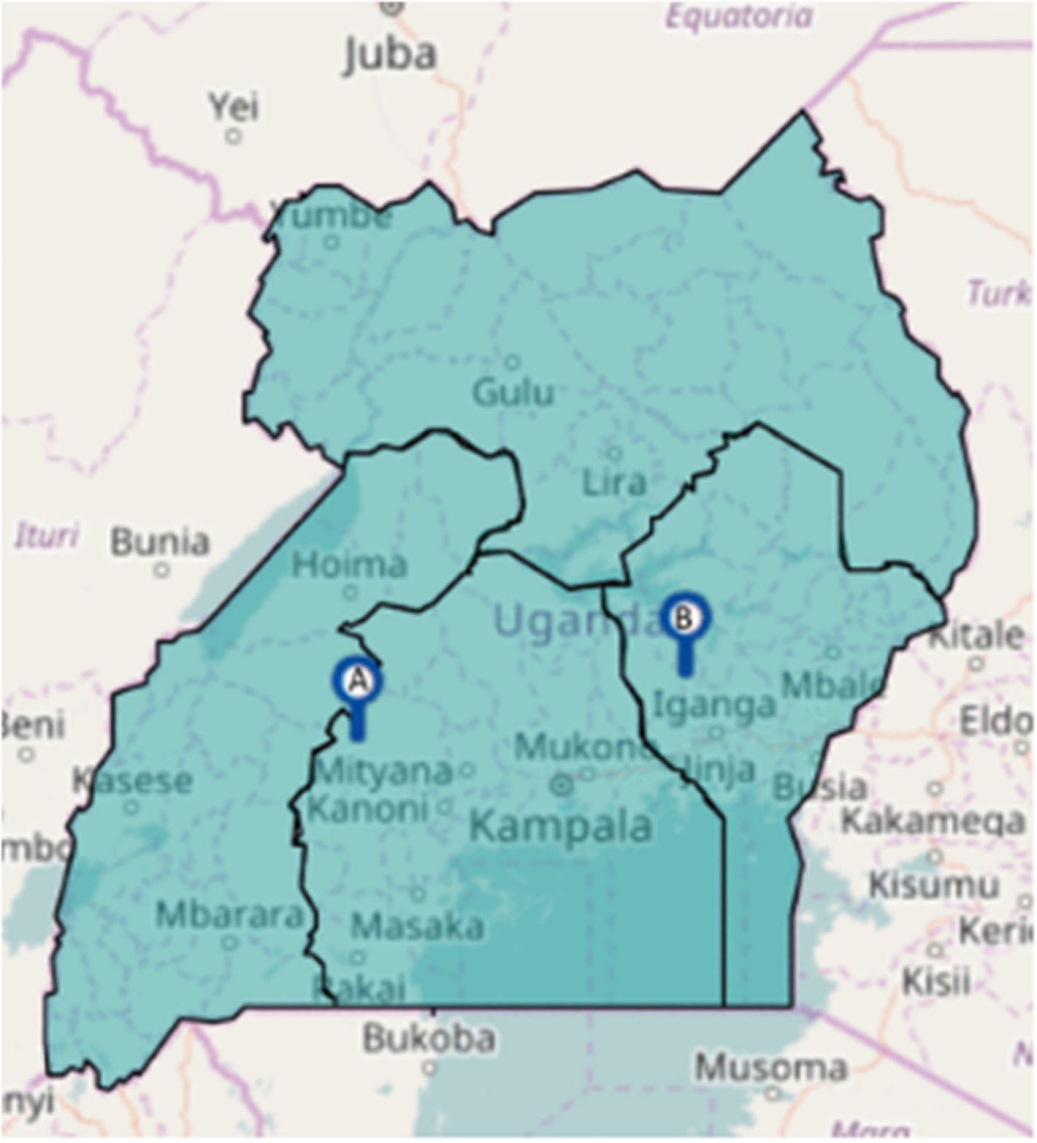
Map of Uganda with enrollment sites shown. **a** Kasambya Health centre III and Mubende Regional Referral Hospital and Mubende District. **b** Nawanyago Health Centre III, Kamuli District. Site map was obtained from prior paper by Beaton et al. [5] where participants evaluated in this study were initially identified. Dr. Beaton provided written permission to use this figure

This was a prospective follow-up study of women previously diagnosed with RHD during a community-based, longitudinal RHD screening study in Central and Eastern Uganda in 2015–2017 [5]. Enrollment sites included two Health Centre III’s (Kasambya and Nawanyago), which are midlevel primary health facilities which provides basic laboratory services, maternity care and inpatient care, and a Regional Referral Hospital (Mubende Regional Referral Hospital), which is a secondary level of care that provided specialized surgical and medical services, basic research and training of nurses and paramedical officers (Fig. 1) [5].

During the initial community-based screening initiative completed by Beaton et al. to identify pregnant women with heart disease, women were counseled on recognition of cardiovascular symptoms and asked to return to the designated healthcare centres for evaluation [5]. These centres were often the most accessible and adequately resourced and for affected women living in nearby regions. If a woman needed to relocate, she was redirected to new centre for required injections.

Our research team completed dedicated follow up visits at these various sites over the course of 2 weeks from March 25 to April 5, 2019. Eligible mothers were contacted by phone, and if not reached, village healthcare workers and village leaders were utilized to attempt in-person contact. Women were invited to their local healthcare clinic on days the research team was visiting and provided with transport refund. Presenting women were counseled on all aspects of the study and follow-up visit and signed written informed consent in their preferred language. Confidentiality was ensured by usage of unique patient de-identifiers maintained on an encrypted list. Consented participants underwent a three-step mixed methods study to include 1) directed structured interview to capture clinical history, Benzathine Penicillin G (BPG) adherence (the standard of care for prophylaxis in definite RHD), and clinical follow-up 2) echocardiogram focused on assessment of the left-sided valves, 3) semi-structured guideline interview to elicit the personal impacts of RHD on participant life, including access to care and reproductive decision making.

The structured postpartum interview guide (see Additional file 1) was partially adapted from that used during initial antenatal care visits of women identified with RHD during pregnancy [5]. This questionnaire elicited specific cardiovascular symptoms that women had also been asked about during their pregnancies. An additional section was developed to assess long term medical care adherence, including type and timing of follow-up visits and adherence to prescribed cardiac medications, as well as individual reproductive choices following the diagnosis of heart disease.

All procedures were approved by the institutional review boards of Mengo Hospital, Children’s National Medical Center, and Cincinnati Children’s Hospital Medical Center. The study was also approved by the Ugandan National Council of Science and Technology.

### Clinical outcomes

To capture clinical outcomes, a research nurse conducted the clinical interview described above. This component was solely based on patient interviews and recall, as detailed medical records for women were unavailable given resource limitations. BPG adherence during our follow up period was defined as good (receipt of > = 80% of prescribed doses) poor (receipt of < 80% of prescribed doses), or non-adherent (not receiving any doses).

### Echocardiographic protocol

A limited echocardiogram (Table 1) was obtained by one of two senior cardiology fellows (SV, AM) who had not previously performed echocardiograms on this cohort and were blinded to their prior diagnoses, using a fully functional portable machine (General Electric VIVID Q, Milwaukee, WI). These echocardiograms were transferred to secure USB in DICOM format and imported into an echocardiography PACS system (Philips ISCV, Best, Holland). Follow-up echocardiograms on all consented post-partum women were compared to their third-trimester echocardiogram from the prior pregnancy, or in cases where a third-trimester echocardiogram was not available, to the latest gestational echocardiogram from the prior pregnancy. A board-certified cardiologist (CS), expert in RHD, graded the follow-up studies as mild, moderate, or severe RHD based on standard AHA/ACC valvular heart disease criteria [7], based on severity at the most affected valve. Echocardiograms were read as stable, improved, or worsened based on a comparative assessment of valvular incompetency or stenosis.

**Table 1:** Echo protocol

**Parasternal long axis views**	**Step 1 - Parasternal long axis view 2-D:** Visualize mitral valve and aortic valve (used for morphological assessment by expert reviewers) and store loop [3 s]. **Step 2 - Parasternal long axis view color Doppler mode, mitral valve:** Place the color Doppler window over the mitral valve and left atrium – note if there is a regurgitant jet (blue) and store loop. **Step 3 - Parasternal long axis view color Doppler mode, aortic valve:** Place the color over the aortic valve – note if there is a regurgitant jet – If the jet takes up more than half the width of the outflow tract, then measure the width and the outflow tract diameter and store loop.
**Apical 4 chamber view**	**Step 4 - Apical 4 chamber view 2D mode:** Obtain image of all 4 chambers, visualizing mitral valve well and store loop. **Step 5 - Apical 4 chamber view color Doppler mode, mitral valve:** Place the color window over the mitral valve and left atrium – note if there is a regurgitant jet – Store loop. Place cursor in the left ventricle through the center of the mitral valve and obtain continuous wave Doppler – freeze and trace to obtain a mean gradient and store measurement. **Step 6 - Apical 4 chamber view color Doppler mode, tricuspid valve:** Place the color window over the tricuspid valve and right atrium – note if there is a regurgitant jet – Store loop. Place the cursor in the right atrium over the tricuspid regurgitation and obtain a CW Doppler attempting to obtain a full envelope of TR. Freeze and measure the maximum velocity/gradient and store measurement
**Apical 5 chamber view**	**Step 7 - Apical 5 chamber view 2D mode:** Visualize aortic valve and store loop. **Step 8 - Apical 5 chamber view color Doppler mode, aortic valve:** Place the color window over the aortic valve and left ventricle, and note if there is a regurgitant jet – Store loop. Place the cursor across the aortic valve and obtain a CW Doppler to obtain three full envelopes for the systolic flow across the aortic valve. Trace all three envelopes to obtain peak and mean gradient across the aortic valve and store image.

### Open-ended interview

A local, trained village health worker, either male or female and of varying education level, conducted a semi-structured guideline interview (see Additional file 1). These village health workers had established rapport with our study participants, as they had worked closely with these women following their diagnosis of RHD during pregnancy. At each clinical follow-up site, workers were briefed by our research team on overall study aims and methods of non-biased qualitative interview skills. The one-time interviews were then conducted privately between the participant and village health worker, lasting up to 45 min. They were led in the participant’s primary language, directed towards understanding barriers to BPG adherence and to maintaining clinical follow-up, as well as eliciting impact of diagnosis on the mother, and community. Interviews were digitally recorded, without additional field notes, and transcribed verbatim into English for subsequent analysis.

### Data analysis

Study data were collected and managed using REDCap electronic data capture tools hosted at Children’s National Medical Center [8]. Quantitative clinical and echocardiographic data were described using frequencies and percentages, or medians with interquartile ranges where appropriate. Fisher’s exact testing was used to compare categorical data on pregnancy and follow-up (MedCalc for Windows, Version 15.1, Ostend, Belgium). Qualitative data were analyzed using qualitative description (QD) methodology [9–11]. Qualitative data was examined by two team members (SV, MM) who independently coded the data, line by line, by the constant comparative methods, identifying patterns and themes [12]. These interviews were then revisited in a series of iterative steps by an independent team member (RS) to confirm the classification of codes and that theoretical saturation had been reached. Data were recorded, coded and analyzed using Microsoft Word (Redmond, WA) and Microsoft Excel (Redmond, WA). The final manuscript was subjected to the COREQ checklist (see Additional file 1) for consolidating criteria for reporting qualitative research [13].

## Results

**Fig. 2:**
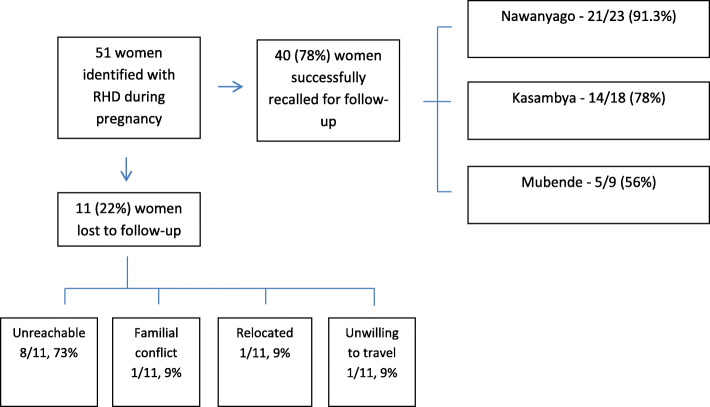
Enrollment flowchart of follow up subjects

The team evaluated 40 (80%) of the original 51 mothers with RHD at a median post-partum time of 2.5 years after delivery (IQR 0.5). During this time, there was no contact between participants and our research team. Of those women not seen, one had died in the postpartum period, related to infectious post-partum complications following a home delivery; seven were unreachable, and three were unable or unwilling to attend the follow-up assessment (Fig. 2). There was no further post-partum clinical information available on the women not successfully recalled, though during their pregnancies there were no noteworthy clinical distinctions in the women lost to follow-up. The major barrier to follow-up evaluation, particularly at Mubende Regional Referral Hospital, was related more to logistical challenges experienced by our outreach, including inability of the local nurses to make successful contact with women in a resource-challenged environment and further distance of women enrolled from the health facility, rather than inherent differences to that particular region or population. Among the 40 women successfully recalled, 35 had not had a subsequent pregnancy, two had delivered another child, and three were currently pregnant (20–36 weeks gestation). The median maternal age during pregnancy was 30.5 years (IQR 12) and 33.7 years (IQR 11.7) at follow up.

### Clinical outcomes

Reported cardiovascular symptoms were similar between pregnancy and follow-up except for syncope, which was more common during pregnancy (5/40 vs. 0/40, *p* = 0.05) (Table 1). Nearly all women (98%) remembered prescription of BPG, but only 30% (12/40) of women had good adherence, 15% (6/40) had poor adherence, and 55% (22/40) were non-adherent. Retention in care showed similarly low rates, with only 30% of women (12/40) having seen a medical provider for heart disease since delivery.

### Echocardiographic outcomes

The median gestational age for pregnancy echocardiogram was 30 weeks (IQR 6.0). Table 2 shows echocardiography findings during pregnancy and one-year follow-up.

**Table 2:** Cardiovascular symptoms at specified follow up visits in women with RHD

Cardiac symptoms	Pregnancy	Follow-up	*p*-value
Palpitations, n (%)	17/40, 43%	18/40, 45%	1.0
Exercise intolerance, n (%)	13/40, 33%	16/40, 40%	0.64
Chest pain, n (%)	13/40, 33%	14/40, 35%	1.0
Shortness of breath, n (%)	8/40, 20%	6/40, 15%	0.77
Swelling, n (%)	6/40, 15%	6/40, 15%	1.0
Syncope, n (%)	5/40, 13%	0/40, 0%	**0.05**
Hemoptysis, n (%)	0/40, 0%	0/40, 0%	1.0
No symptoms, n (%)	13/40, 33%	18/40, 45%	0.36

Significant echocardiographic improvement between pregnancy and follow-up were found in nine of 40 women (23%). Degree of mitral regurgitation was the most dynamic finding. Reduction in the grade of mitral regurgitation at follow-up was seen in nine women: two women with mild mitral regurgitation in pregnancy improved to non-pathologic mitral regurgitation and seven women with moderate/severe mitral regurgitation in pregnancy (one with concurrent stable moderate aortic insufficiency) improved to mild mitral regurgitation. No woman worsened. Sub-analysis of the three patients with repeat pregnancy at 1 year follow up showed stable mitral regurgitation and stable moderate mitral stenosis (improvement to mild in the post-partum period) when compared to initial pregnancy echocardiogram.

### Barriers to care

During clinical intake, women most commonly cited financial limitations including transportation and cost of medication as the major barrier to BPG adherence and retention in care. Additionally, lack of providers and distance to higher level facilities limited access to medical care following diagnosis and made monthly BPG injections difficult to maintain (Table 3).

**Table 3:** Comparison of echocardiographic findings in women with RHD during pregnancy and at follow-up

	Pregnancy (*n* = 40)	Follow-up (*n* = 40)	*p*-value
RHD severity			
Mild RHD	20	24	0.50
Moderate/Severe RHD	20	14	0.26
Normal (non-pathologic MR)	0	2	0.49
Mitral regurgitation			
Trivial/Mild	17	21	0.50
Moderate/Severe	17	11	0.24
Mitral stenosis			
Mild	0	0	n/a*
Moderate	1	1	n/a*
Severe	0	0	n/a*
Aortic insufficiency			
Mild	3	3	n/a*
Moderate	2	2	n/a*
Severe	0	0	n/a*

*Not calculated secondary to low numbers

### Personal impact of RHD

Three major themes emerged from interviews (Table 4): 1) social determinants of health [14] negatively affecting healthcare, 2) RHD diagnosis negatively affecting female societal wellbeing, 3) central role of spouse in medical decision making (Tables 5 and 6).

**Table 4:** Barriers to care

	BPG, n (%)	Retention in medical care, n (%)
Transportation cost/availability	18/40 (45%)	21/40 (53%)
Drug cost	12/40 (30%)	9/40 (23%)
Lack of healthcare workers	9/40 (23%)	9/40 (23%)
Distance	3/40 (8%)	6/40 (15%)
Fear of injection	6/40 (15%)	3/40 (8%)
Misunderstanding or lack of understanding of disease process	–	3/40 (8%)
Conflict with husband/family	3/40 (8%)	3/40 (8%)
Drug availability	1/40 (3%)	2/40 (5%)
Other	4/40 (10%)	1/40 (3%)

**Table 5:** Qualitative interview themes

Social determinants negatively affecting healthcare	RHD diagnosis negatively affecting female societal wellbeing	Central role of spouse in medical decision making
- Poverty	- Stigmatization	- Personal desire
- Health care access	- Impact on daily activities	- Spousal pressure
- Health literacy	- Adverse personal emotions	- Cardiovascular symptoms in pregnancy
- Gender

**Table 6:** Exemplar quotations illustrating themes from qualitative interviews

Social determinants	Poverty/Healthcare access: *“I am requesting that the health workers continue to treat us and also to provide us with some transport since we stay very far, travel long distances yet we are very poor”* Female gender role: *“As a woman, there is too much work to do no matter whether you are sick or not, you have to carry on with the duties like digging.”* Health literacy: *“I used to sell charcoal, we could go and buy it from far places, climb those Lorries, and I think that is where I got this disease from”* *“I would like to stop giving birth but am afraid of which family planning method to use and from the rumors that I hear, with this heart problem how will it treat me”*
Central role of spouse in medical decision making	*“My husband told [the healthcare worker] that we had come here to be helped and if she found a disease and she could treat it, she should go ahead as long as it was free because we did not have any money for that. And when the health worker agreed, I started on treatment.”* *“My husband and family felt bad and they thought I was going to die since we do not have money to treat such a complicated disease”* *“I told my husband after I went back home about my diagnosis, he was scared but as a man, he had to keep strong”* *“I don’t find any problem telling the community because every time you hide your illness, you don’t get the help you need. I like it because in case of any attack, people around can help you call your husband”*
RHD diagnosis negatively affecting female societal wellbeing	*“I do not want the community members to know because they will stress me and stigmatize me thinking I am HIV positive”* *“I can no longer keep up with my colleagues because the speed at which I do things has reduced so much due to my heart condition”* *“There are some things I used to do that I can no longer do as my body has weakened like digging, carrying heavy stuff”* *“I don’t have hopes of getting pregnant because I am worried of the complications which might result from the heart disease which I already have”*

### Social determinants negatively affecting healthcare

Many participants self-identified as living in poverty and attributed their poor medical adherence to financial barriers with respect to cost of medications and transportation. Nearly all participants believed their healthcare, and that of other affected women, would be improved if healthcare services were closer or transportation less expensive. As described by one participant, “*I have taken a year off treatment and I do not feel so well. I will try to take my treatment as long as the medicine is not so expensive. The biggest challenge is the money for transport*.”

While few participants mentioned their gender outright in describing their experience with RHD, many alluded to their roles in the family and community as females. The undertone that women’s health would not or could not receive priority over other family needs was implicit in many participants’ remarks, and some mentioned lack of control in allocation of finances as dictated by the husband. Women expressed variable degrees of choice in reproductive decision making despite possible maternal risk, with one representative woman stating, “*I am ready to stop having children. My husband would agree with me, but the only problem is that he wants a male child. He said that once we get one, I can go and get my tubes cut*.”

The interviews also revealed a gap in health literacy among affected women and among their healthcare providers. Women expressed misconceptions about the etiology of RHD and the role of BPG and concerns about the lack of knowledge among local healthcare workers to care for their disease. One woman summarized, “*We have nowhere to get the drugs and when you approach some health workers, they are not aware of the disease and we got lost and mixed up in the whole system*”.

### RHD diagnosis negatively affecting female societal wellbeing

Participants commonly described societal hardships associated with the diagnosis of RHD. Fear of stigmatization led some to withhold health information, however most women shared their RHD diagnosis and health information with their immediate family. Many others shared their health information with the greater community in order to engender increased support. As described by one mother. “*I don’t find any problem telling the community because every time you hide your illness, you don’t get the help you need. I like it because in case of any attack, people around can help you call your husband*”. On the contrary, others were met with stigma on sharing with the community and many were presumed to have HIV based on need for ongoing medical care and chronic injectable BPG. As one woman stated, “*The community laughs at you saying you got HIV*”.

Isolation from family and community was reported as a contributor to detrimental maternal emotions, with a majority of women describing fear, worry, and adverse perception of self-worth after learning of their diagnosis. Women also reported community bias based on the perception of their ability to contribute with manual labor. As reported by one woman, “*They advised my husband to get another woman because according to them, someone with a heart problem cannot do most of the work a woman should. I really hated my neighbors and other village members knowing about my heart issues*”.

### Impact of spouse in medical decision making

Women consistently reported that their spouse served as either a facilitator or barrier in RHD management, including the decision to have more children. Some women described the emotional and financial support provided by their husbands, “*When I went back home, I told my husband about it. He was happy and encouraged me to go for treatment*”. Others reported that RHD diagnosis created tension in the family with some husbands opting to take on another wife, “*I have no problem continuing with my treatment, my only problem is my husband. Since he got a new wife, he complains when I ask for money, he says I ask for money all the time*”. Many of these tensions centered around the cost of medical treatment, with many women expressing financial dependency, “*Continuing with treatment and paying for it will depend on how much it costs because since I depend on my husband, he will be the one to determine whether I can afford the treatment or not*.”

Reproductive decision making was also a central theme, intimately linked to spousal dependency. While several women cited their heart disease as a reason to avoid future pregnancies, “*This is the last child am having because according to the condition of my heart and what I go through when am pregnant*. *.*. *I cannot get pregnant again*”, external drivers such as spousal desire and community pressure for more children appeared more frequently, “*I tried family planning, but my husband refused so I had to stop*.”

## Discussion

Maternal RHD poses an immediate risk to the pregnant woman and to her fetus’s and her newborn’s wellbeing. However, for those who survive, the impact of an RHD diagnosis extends far beyond gestation with ongoing clinical and personal consequences. For the first time, a prospective echocardiographic screening study for early RHD diagnosis has allowed follow-up of a population of mothers newly diagnosed with RHD in Africa. Our data demonstrate that many women experience worsening of valve disease during pregnancy, that most struggle to maintain adequate secondary BPG prophylaxis and access to care following diagnosis, and that a woman’s ability to access care and maintain societal wellbeing is impacted by social determinants of health and spousal dependence for medical decision making.

Our data clearly show that valvular heart disease, as has been well-established [15–17], worsens in pregnancy. While ideally women should know their RHD status prior to pregnancy, pregnancy may also provide an opportunity for primary RHD diagnosis. Moreover, though the majority of our population included pathological mitral regurgitation as opposed to mitral stenosis, pulmonary arterial hypertension would be an interesting point for future study during long term follow-up of these patients. In this cohort, nine women showed worsening of valvular disease during pregnancy, which improved following delivery. Identification of women such as these, relatively early in the RHD spectrum, may provide the most benefit from BPG initiation, preventing worsening of heart disease and protecting them in future pregnancies.

The data from our post-partum clinical questionnaire (Table 2) also highlight the challenge of making a clinical diagnosis of RHD during pregnancy as symptoms of heart failure show substantial overlap with common symptoms of pregnancy (i.e. shortness of breath, fatigue, exercise intolerance, lower extremity swelling). Further research is needed to compare symptomatic profiles between pregnant women with and without rheumatic heart disease in this population to uncover if more specific predictors or patterns exist.

Women in our cohort showed a disappointing rate of adherence to BPG prophylaxis, the cornerstone of RHD management. The same local nurses who were involved in patient care visits during the initial study were directly involved in connecting with patients for this follow-up study, therefore continuity of care had been previously established. Our hope was that treatment adherence would better than we found here. Previous data from Uganda has shown higher adherence among children and adults diagnosed at a tertiary facility [18–20] suggesting women detected through active case finding, or screening, may be less likely to be retained in care. Of note, slightly more than half of women were completely non-adherent or had received zero doses of BPG in the previous 12 months. This is consistent with data on the RHD cascade of care in Uganda, which showed that those who can maintain retention in medical care often receive high percentages of injections, while those who do not have continuity of care receive none [21].

Innovative strategies and healthcare systems strengthening are needed to promote RHD awareness and improve adherence rates. Our study elicited gaps in healthcare literacy among local healthcare workers. Extension of shared RHD learning into community education across the health profession spectrum may spread knowledge, dispel medical mythology, and drive advocacy and policy change [22]. In Australia, positive patient-staff interactions [23, 24] were shown to be the most powerful driver of adherence. In New Zealand, dedicated RHD-nurses and engaged community health workers results in high adherence rates [25], an approach that has also resulted in highly adherent pediatric patients in some parts of Uganda [21]. Leveraging technology could also play a pivotal role, with young people in Fiji identifying mobile phone reminders as the most useful form of healthcare follow-up [26]. Ongoing work in Uganda through the American Heart Association’s Strategically Focused Research Networks [27] to provide national data on the health-system response to RHD may provide additional interventions to improve adherence in this context.

The qualitative themes identified in our study are corroborated by another study of 75 Ugandan women with RHD, where spousal control over reproductive decision making, and stigmatization by society on the basis of limitations imposed by heart disease [6] emerged as major themes. Similarly, in Rwanda, Kenya, and Ghana family planning has been found to be impacted most by societal pressure, gender roles, and misconceptions as well as cost [28–31]. These powerful drivers suggest a community-engaged approach to family planning interventions and RHD education is essential to aid understanding and acceptance of pregnancy avoidance for those at highest risk from RHD and other non-communicable diseases.

Overall, this follow-up study reconnected care between local nurses/ village health workers and affected women to help establish community-based connection and improve care adherence. Further effort is undoubtedly needed to continue to address the challenges faced within this population.

## Conclusion

In sum, our findings suggest that diagnosis of RHD during pregnancy has both long-term clinical and psychosocial impact. When considering screening programs, the benefits of early diagnosis (improved outcomes, long-term prophylaxis to prevent heart disease advancement) must be balanced by a community-engaged approach that mitigates secondary harm. Women diagnosed through screening carry a particularly high risk of loss to follow-up, and innovative solutions are needed to effectively delivery secondary prophylaxis and retain women in care. Additionally, community, patient, and provider education may result in improved linkage to care, outcomes, and patient empowerment. Further research is needed to determine how to best support women who face a new diagnosis of RHD, and to determine the role of screening echocardiography in high-risk settings.

### Limitations

Limitations of this study include restricted sample size and challenges of recalling all previously diagnosed women with RHD in a limited resource environment. Additionally, the subjective recall bias inherent to both the clinical questionnaires and qualitative interviews must be acknowledged.

## Supplementary information

**Additional file 1.**

## Data Availability

The datasets generated and analyzed in this study are available from the corresponding author on reasonable request.

## Abbreviations

LMICs: Low and Middle Income Countries
RHD: Rheumatic Heart Disease
BPG: Benzathine Penicillin G
ACC: American College of Cardiology
PACS: Picture Archiving and Communication System
AHA: American Heart Association
