# Supplementary material for: The personal and clinical impact of screen-detected maternal rheumatic heart disease in Uganda: a prospective follow up study
Source: BMC Pregnancy Childbirth. 2020 Oct 9;20:611. doi: 10.1186/s12884-020-03189-z (PMC7547429; doi:10.1186/s12884-020-03189-z)
Supplement: Supplementary file 1 — Additional file 1. [file 12884_2020_3189_MOESM1_ESM.zip › Post Partum Clinical QuestionnaireR3.docx]

**Post-Partum Clinical Questionnaire**

ITW #:___________________ Date:______________________

SUBJECTIVE FINDINGS

Cardiovascular Symptoms: Shortness of breath at rest, swelling, syncope, hemoptysis, palpitations, limited exercise tolerance, chest pain.

Please expand on any positive symptoms:

MEDICAL FOLLOW-UP HISTORY

Since your delivery have you seen any health provider for care of your heart: Yes No

If yes, who have you seen?

Were you referred to a higher-level facility (as compared to an HCIII): Yes No

What is the highest-level facility where you have had a cardiac assessment?

Do you have another follow-up for your heart scheduled? Yes No

Please describe any barriers to follow-up for your heart:

MEDICATION HISTORY:

Prescribed Secondary Prophylaxis: Yes No

If yes, form: IM PO

If yes, date prescribed:

If yes, # of injections received (review BPG log):

If no, when and why did you stop secondary prophylaxis?

Please describe any barriers to receipt of secondary prophylaxis:

Prescribed additional cardiovascular medications: Yes No

If yes, which medications were you prescribed?

Have you continued these medications post-partum?

If no, why not? When did you stop?

Please describe any barriers to obtaining or taking these medications:

CONTRACEPTION USE

Were you counseled about the need for future contraception / avoidance of pregnancy: Yes No

Comments:

Have you actively tried to prevent another pregnancy since your diagnosis?

Yes No

Why or why not?

Please expand on your decision to continue attempting or preventing pregnancy.
