# Supplementary material for: The personal and clinical impact of screen-detected maternal rheumatic heart disease in Uganda: a prospective follow up study
Source: BMC Pregnancy Childbirth. 2020 Oct 9;20:611. doi: 10.1186/s12884-020-03189-z (PMC7547429; doi:10.1186/s12884-020-03189-z)
Supplement: Supplementary file 1 — Additional file 1. [file 12884_2020_3189_MOESM1_ESM.zip › Qualitative Interview GuideR3.docx]

**Qualitative Interview Guide**

We know that being diagnosed with heart disease during your pregnancy may have come as a shock. It is hard to carry this kind of diagnosis and to think about what it might mean for your life and your family. We are interested in helping women in the future who are found to have heart disease during pregnancy and wanted to explore more with you what this has meant to you, your family, and your community. These questions are meant to start our conversation, but please add anything else you want healthcare provider to know about being diagnosed with heart disease during pregnancy. There are no right answers. Your answers are all confidential.

What was it like to receive a diagnosis of heart disease during your pregnancy?

(Follow-up if needed). *What did you think? What did you feel?*

With whom did you share this diagnosis?

(Follow-up if needed). *What was it like to share the diagnosis? What was the reaction of the person/people you told? Are there people whom you don’t want to know about your diagnosis? Why?*

How has your diagnosis affected your immediate family?

How has your diagnosis affected your community?

Has your diagnosis changed how you feel about future pregnancies?

What would you like to share with doctors and nurses who may diagnose pregnant women with heart disease in the future?
